# Improving treatment outcomes for leprosy in Pernambuco, Brazil: a qualitative study exploring the experiences and perceptions of retreatment patients and their carers

**DOI:** 10.1186/s12879-021-05980-5

**Published:** 2021-03-19

**Authors:** Divya Khanna, Gilles de Wildt, Luiz Antonio Miranda de Souza Duarte Filho, Mitali Bajaj, Jo Freda Lai, Esme Gardiner, Andrea Maia Fernandes de Araújo Fonseca, Antje Lindenmeyer, Patrícia Sammarco Rosa

**Affiliations:** 1 Medical School, College of Medical and Dental Sciences, University of Birmingham, Birmingham, UK; 2 Institute of Clinical Sciences College of Medical and Dental Sciences, University of Birmingham, Birmingham, UK; 3 Universidade Federal do Vale do São Francisco, Pernambuco, Brazil; 4 Serviço de Infectologia, Petrolina, Pernambuco Brazil; 5 School of Nursing, University of Birmingham, Birmingham, UK; 6 Instituto Lauro de Souza Lima, São Paulo, Brazil

**Keywords:** Leprosy, Patient, Carer, Experiences, Perceptions, Qualitative, Brazil

## Abstract

**Background:**

Brazil has a high leprosy burden and poor treatment outcomes (TOs), manifesting in high relapse rates. Pernambuco, an impoverished Brazilian state suffering notable geographical health inequalities, has ‘*hyperendemic’* leprosy. Although current literature identifies barriers and facilitators influencing leprosy treatment compliance, inadequate investigation exists on other factors influencing TOs, including carers’ roles and psycho-dermatological impact. This qualitative study explores experiences and perceptions of leprosy patients and their carers in Pernambuco, Brazil; to identify location-specific factors influencing TOs, and consequently inform future management.

**Methods:**

27, semi-structured, in-depth interviews were conducted with 14 patients and 13 carers. Participants were recruited using maximum variation and snowball sampling from three clinics in Petrolina, Pernambuco. Transcripts and field notes from both participant groups were separately analysed using conventional thematic and deviant case analysis. The University of Birmingham Internal Research Ethics Committee and Instituto Lauro de Souza Lima provided ethical approval.

**Results:**

Two homologous sets of four, primary, interdependent themes influencing leprosy TOs emerged: *‘personal factors’*; *‘external factors’*; *‘clinical factors’*; and *‘the healthcare professional (HCP)-patient-carer relationship’*. Poor participant knowledge and lack of symptomatic relief caused patients to distrust treatment. However, because participants thought HCP-led interventions were vital for optimal TOs, patients were effectively persuaded to adhere to pharmaceutical treatments. High standard patient and population education facilitated treatment engagement by encouraging evidence-based medicine belief, and dispelling health myths and stigma. Healthcare, on occasions, was perceived as disorganised, particularly in resource-scarce rural areas, and for those with mental health needs. Participants additionally experienced incorrect/delayed diagnoses and poor contact tracing. Leprosy’s negative socio-economic impact on employment – together with stigma, dependency and changing relationships – caused altered senses of identity, negatively impacting TOs. Better dialogue between patients, HCPs and carers facilitated individualised patient support.

**Conclusion:**

This study highlights the importance of: effective evidence-based leprosy education; communication between HCPs, patients and carers; state-funded support; and healthcare resource distribution. These findings, if prioritised on governmental scales, provide the valuable insight needed to inform location-specific management strategies, and consequently improve TOs. Future research should evaluate the effectiveness of these implementations. Failure to address these findings will hinder regional elimination efforts.

**Supplementary Information:**

The online version contains supplementary material available at 10.1186/s12879-021-05980-5.

## Background

Leprosy is a chronic, highly stigmatised, neglected tropical disease, caused by *Mycobacterium leprae,* with dermatological, neurological and ophthalmic complications [1–3]. While leprosy’s transmission risk is low (with only 2–5% of those exposed becoming symptomatic), its slow progression poses permanent disability risks [2]. The World Health Organisation (WHO) has been instrumental in global leprosy elimination campaigns, through their endorsement of a freely available, antimicrobial-based *‘multidrug therapy’* (MDT) for patients of endemic countries [4]. Since 1981, over 16 million patients have been cured globally through these WHO efforts [5–7]. However, despite MDT easing leprosy’s global burden notably, the infection remains endemic in several low- and middle-income countries worldwide [8].

In 2018, Brazil, a middle-income South American nation, reported the second highest leprosy incidence globally (after India and Indonesia); responsible for 13.7% of worldwide cases [8, 9]. Brazil’s leprosy incidence rose by 14% between 2015 and 2018, despite initially declining post-2009, indicating a recently worsening endemic [8]. Further scaling the problem, in 2018, Brazil also reported the second highest number of both ‘*grade two disabilities*’ (visible deformities/severe visual impairment secondary to leprosy) and ‘*retreatment cases*’ (patients re-diagnosed with leprosy after receiving treatment previously) worldwide [8, 10].

Pernambuco, a state in North-East (NE) Brazil where 27.17% of all households live in poverty, has ‘*hyperendemic*’ leprosy status. Healthcare access in Pernambuco is *‘poor’*, due to geographical inequalities present in Brazil’s state-funded, primary care focussed healthcare system, and high costs associated with better resourced private healthcare [10–17]. In 2010, Pernambuco had almost double the national leprosy prevalence (3.01/10,000 inhabitants, compared to 1.62/10,000 nationally), cure rates 3% lower than national rates, and the highest percentage of MDT-discontinuing patients countrywide (8.2%) [18, 19].

These statistics indicate poor treatment outcomes (TOs) in Pernambuco, particularly in terms of cure, symptom improvement, quality of life, coexisting illness, and healthcare service discharge [18]. The interdependent implications of poor TOs go beyond mere statistics. Firstly, poor TOs act alongside the stigma and negative religious connotations associated with leprosy in Brazil, further impacting patient quality of life, mental and physical health, and socioeconomic status [20–22]. Furthermore, while antimicrobial resistance (AMR) currently causes only 5% of Brazil’s retreatment cases, poor TOs encourage this percentage, and the global AMR concern, to rise [23–25]. Poor TOs in Pernambuco also increase patient infectiousness; promoting disease transmission and hindering regional WHO elimination efforts [10].

As for any disease, improving leprosy TOs in Pernambuco requires a location-specific insight into the population’s experiences, perceptions and beliefs of leprosy and its treatment [26]. Literature on other diseases shows carer support has a symbiotic relationship with TOs; therefore, their views should be appreciated alongside those of patients [27–31]. To gauge the depth of evidence currently available, a MEDLINE literature search for English and Portuguese language literature, or literature with English and Portuguese language abstracts, was conducted. Because experiences and perceptions require probing and prove difficult to quantify, only qualitative literature was searched [29]. The following search terms were used: ‘leprosy AND therapy’ AND ‘patient OR family OR carer’ AND ‘attitude OR perception OR patient dropout OR noncompliance OR relapse’. Results were limited to those published post-2009, as this year limit coincides with the time frames of the most recently published WHO global leprosy statistical analysis [8]. Reference lists of identified papers were searched for ‘grey’ literature, which was not previously located through the systematic literature searches.

Six studies were identified [27, 32–36], of which one was found when searching for ‘grey’ literature [36]. One systematic review (SR) of 20 qualitative studies published between 2005 and 2013 identified numerous facilitators and barriers of treatment compliance, which they grouped into two categories to form a theoretical framework: ‘*personal factors*’ (sub-divided into ‘*life quality*’, *‘socio-economic factors’*, and *‘cultural needs’*) and *‘medical factors’* (sub-divided into *‘treatment regimen’* and *‘health services’*) [32]. However, the five further studies found facilitators and barriers of treatment compliance that are neither mentioned in, nor can be grouped into, the SR’s theoretical framework, including concepts surrounding: quality of knowledge on leprosy; importance for one’s own health; health beliefs; personality and behaviour; and experiences of stigma [27, 33–36]. This indicates that the SR’s theoretical framework is not comprehensive.

There are further limitations to these studies beyond the lack of a comprehensive theoretical framework. Some studies used qualitative questionnaires, which restricted the depth and breadth of participant responses [27, 32, 35]. Others recognised that their findings are not transferable, and therefore proposed the need for further, location-specific research, in order to effectively inform local healthcare policy-making [27, 32–34, 36]. Brazil, a nation with unique socio-economic profiles, cultural attitudes, political climates and healthcare delivery structures which vary considerably by state and municipality, would benefit from this, as factors such as: the quality of leprosy-related knowledge; side effect impact; health beliefs; and religion have only been explored in non-Brazilian participants [18, 32]. Additionally, findings of the last published Brazilian study may no longer be relevant, as viewpoints may have shifted with the recently increasing national leprosy incidence [8, 32]. The research-deprived NE region would also benefit from location-specific research, as the geographical density of leprosy research in Brazil is not distributed proportionally to the geographical density of disease; with most studies conducted in the less affected, wealthier South-East region [37]. No literature exists on carers, despite their impact on TOs documented in studies of other diseases [28, 31, 38]; or on retreatment patients, who form an information-rich subgroup of leprosy patients with first-hand experiences of disease relapse, and therefore poor TOs [39]. Finally, apart from one Portuguese study which focussed on stigma [36], the present literature focuses primarily on facilitators and barriers of medication compliance. Novel research is therefore required, as medication compliance and stigma are only two of the factors influencing TOs [31, 38]. These evidence gaps collectively justify the WHO’s decision to make investigation into factors influencing leprosy TOs their research priority [23].

Overall, the literature search indicates poor understanding on the barriers and facilitators of leprosy TOs in Pernambuco. This study, part of a wider study titled, [40] aims to address the evidence gap alongside the WHO research priority, by exploring retreatment patients’ and carers’ experiences, perceptions and beliefs of leprosy and its treatment in Pernambuco.

## Methods

### Design

A qualitative, in-depth interview study design, employing semi-structured topic guides, was used for a holistic, profound exploration of the study aim. While quantitative methods have more rigour and yield generalizable, statistically significant numeric data, this qualitative design was best suited to gather rich data on the complex, multifaceted, sensitive topics being explored [41–45]. Focus groups presented issues of confidentiality, unequal participant input, and peer influence, and therefore were not chosen [44].

### Setting

This study was conducted in Petrolina: a densely populated, agricultural city of Pernambuco, NE Brazil, with a 2010 population of 293,962 [46]. Petrolina was chosen due to similar and therefore representative demographics to Pernambuco [18, 46]. Three, free, state-funded clinics, all part of the Sistema Único de Saúde (SUS), Brazil’s publicly funded healthcare system, were used to identify patients: *Unidade Básica de Saúde Miguel de Lima Durando*, *Unidade Básica de Saúde Gildevania de Oliveira Silva*, and *Serviço de Infectologia de Petrolina*. The two former sites were rurally-located, while the latter was urban.

### Sampling and recruitment

DK, AF, LD and PR used maximum variation purposive sampling to intentionally identify information-rich patients from clinic registers [47]. Snowball sampling enabled interviewees to identify further study participants, and was the primary method to identify carers [48]. Participants were selected based on the following eligibility criteria: a) leprosy retreatment patient, or such a patient’s carer; b) residing in Petrolina; c) aged over 18 years old; and d) fluent in Portuguese or English. Participants were excluded if they: a) had illnesses affecting their ability to conduct a meaningful interview; b) could not provide informed consent; or c) felt interviewing would cause significant harm/stress to them. After each interview, participant demographics (including occupation, age, gender, socioeconomic status and urban/rural residence) were discussed amongst the study team, to identify demographics missing from the sample. To facilitate maximum variation and increase validity, individuals with the identified missing demographics were sought in further sampling, [47] until data saturation was achieved. For comparative purposes, balanced numbers of a) patients and carers, and b) urban and rural participants were recruited.

Recruitment was undertaken in February and March 2020. DK, AF, LD and PR approached eligible participants and explained the study aims and design using participant information sheets. Participants were made aware that partaking was voluntary and would not impact healthcare professional (HCP) care provisions.

### Data collection

Semi-structured, one-to-one, face-to-face interviews were guided by separate topic guides, formulated uniquely for the purpose of this study, for patients and carers (Tables 1 and 2) [see Additional File 1]. The topic guides incorporated factors influencing TOs in previous literature; factors the study supervisors deemed important; and Patton’s six interview questions (behaviour/experience, opinion/belief, feelings, knowledge, sensory, background/demographic) [49]. The semi-structured format permitted flexibility for the discussion of ideas unanticipated by study personnel (for new theme synthesis), whilst enabling interviewers to re-orientate discussions if participants digressed [50, 51]. Pilot interviews, consisting of open, semi-structured questions, were conducted on Day 1 to overcome translational difficulties and allow topic guide familiarisation across the study team. The topic guides were iteratively revised between interviews using the constant comparative method; incorporating questions on unanticipated ideas until no new ideas surfaced, indicating data saturation [52].

**Table 1:** Topic guide for patient participants

Topic	Subtopics
Questions exploring personal factors	▪ Knowledge about leprosy and treatment ▪ Beliefs about leprosy transmission and cure ▪ Experiences of restarting treatment ▪ Importance of health ▪ Leprosy manifestations ▪ Medication side effects ▪ Medication compliance and coping mechanisms ▪ Psychological impact
Questions exploring external factors	▪ Employment and future aspirations ▪ Experiences of diagnosis ▪ Access to healthcare ▪ Importance of HCP ▪ Contact tracing
Questions exploring support network	▪ Sharing diagnosis with community ▪ Stigma ▪ Family and social circle ▪ Religion

**Table 2:** Topic guide for carer participants

Topic	Subtopics
Questions exploring personal factors	▪ Knowledge about leprosy and treatment ▪ Beliefs about leprosy transmission and cure ▪ Experiences of patient restarting treatment ▪ Importance of health ▪ Leprosy manifestations in the patient ▪ Medication side effects and coping mechanisms ▪ Medication compliance ▪ Psychological impact on self and carer
Questions exploring external factors	▪ Experiences of carer role ▪ Employment and future aspirations ▪ Access to healthcare ▪ Importance of HCP ▪ Contact tracing
Questions exploring support network	▪ Sharing diagnosis with community ▪ Stigma ▪ Family and social circle ▪ Religion

Interviews were conducted in Portuguese by PR and LD. Although it is appreciated that using one interviewer is preferable, PR, LD and DK compared questioning styles and discussed issues arising from pilot interviews to ensure interviewing technique similarity.

Informed written/thumbprint consent was obtained from all participants before interviewing, which took place in private consultation rooms at the three aforementioned clinics, or at participants’ homes. Interviews were audio-recorded on password-protected, encrypted devices, with DK writing down field observations immediately after interviews. LD transcribed interviews into English. A random sample of three transcripts were checked by an independent translator to gauge translation quality. Minimal, grammatical changes were made. Participation withdrawal was permitted at any point up to 48 h post-interviewing, to reduce negative impact on the constant comparative method [50, 52].

### Data analysis

Analysing interview data ensued alongside its collection iteratively using the constant comparative method, [52] which determined the point of data saturation. Provisional themes emerging from this were discussed with PR and LD to gain a more culturally astute insight, minimising risks of cross-cultural bias [53]. Conventional thematic and deviant case analyses were conducted by DK after interviewing to produce accurate reflections of the participant voice [54–57]. These analyses were suitable for the study due to absent pre-existing theoretical frameworks, and their ability to yield rich narratives on the most pertinent themes influencing TO [55]. Braun and Clarke’s six step guide, Saldana’s two step cycle and NVivo 12 software were used to systematically approach data coding [56, 57].

JL and MB triangulated 10% of transcripts to introduce unbiased, fresh perspectives on coding, and increase validity [58]. Triangulated data was compared with DK’s coding to increase credibility, [58] with discrepancies and unexpected findings facilitating theme revision. Themes were reviewed by the study personnel to ensure interpretations represented the yielded data, thereby reducing bias associated with a single analyst. Member validation was not practiced due to study time constraints. DK appreciated the impact of researcher bias throughout analysis as part of a reflexive approach [59]. Findings were reported with consideration of the Consolidated Criteria for Reporting Qualitative Research [60].

### Ethical considerations

The study was approved by the Instituto Lauro de Souza Lima ethics committee, Brazil on 11/12/2019 (3.746.443), and the University of Birmingham Internal Research Ethics Committee, United Kingdom on the 06/01/2020 (IREC2019/1636664)**.** Data will be stored for ten years according to the University of Birmingham data protection policy and the Data Protection Act, 2018 [61, 62].

## Results

28 interviews were conducted in February and March 2020. Tables 3 and 4 summarise patient and carer participant demographics, respectively. One interview (011 – Patient) was discarded due to poor recording quality, leaving 27 usable interviews in total. Interviews lasted on average for 20 min, ranging in length from seven (013 – Carer) to 46 min (026 – Patient). All carers were family members of patients. No participants withdrew from the study.

**Table 3:** Summary of patient participants’ demographics

Identifier	Clinic type	Age bracket	Education	Gender	Job	Number of people in household	Consent type
001	Urban	35 ≤ x < 45	Incomplete elementary school	Female	Unemployed	4	Written
002	Urban	45 ≤ x < 55	Incomplete elementary school	Male	Unemployed	5	Thumbprint
004	Urban	65 ≤ x < 75	No formal education	Male	Farmer	3	Thumbprint
005	Urban	35 ≤ x < 45	Completed elementary school	Male	Public agent	2	Written
009	Urban	35 ≤ x < 45	Incomplete elementary school	Male	Self-employed	9	Written
010	Urban	35 ≤ x < 45	Completed elementary school	Male	Farmer	3	Written
012	Rural	75 ≤ x < 85	Incomplete elementary school	Female	Housewife	1	Written
016	Rural	25 ≤ x < 35	Completed high school	Female	Housemaid	5	Written
018	Rural	45 ≤ x < 55	Completed elementary school	Male	Self-employed	3	Written
019	Rural	35 ≤ x < 45	Completed elementary school	Female	Unemployed	5	Written
021	Rural	35 ≤ x < 45	University education	Female	Unemployed	2	Written
022	Rural	65 ≤ x < 75	Completed elementary school	Male	Retired	2	Written
026	Urban	45 ≤ x < 55	Completed high school	Female	Housewife	5	Written
027	Urban	55 ≤ x < 65	Incomplete elementary school	Male	Unemployed	9	Written

**Table 4:** Summary of carer participants’ demographics

Identifier	Clinic type	Age bracket	Education	Gender	Job	Number of people in household	Consent type
003	Urban	35 ≤ x < 45	Incomplete elementary school	Female	Housewife	7	Written
006	Urban	35 ≤ x < 45	Completed high school	Male	Self-employed	1	Written
007	Urban	45 ≤ x < 55	Completed elementary school	Female	Farmer	2	Written
008	Urban	65 ≤ x < 75	No formal education	Male	Retired	2	Thumbprint
013	Rural	55 ≤ x < 65	Completed elementary school	Male	Truck driver	5	Written
014	Rural	35 ≤ x < 45	Completed elementary school	Male	Mechanic	5	Written
015	Rural	55 ≤ x < 65	Incomplete elementary school	Female	Housewife	5	Written
017	Rural	25 ≤ x < 35	Incomplete elementary school	Female	Hairdresser	3	Written
020	Rural	55 ≤ x < 65	No formal education	Female	Housewife	2	Thumbprint
023	Rural	18 ≤ x < 25	Completed elementary school	Female	Unemployed	3	Written
024	Urban	55 ≤ x < 65	Incomplete elementary school	Female	Housewife	3	Written
025	Urban	35 ≤ x < 45	University education	Female	Estate agent	4	Written
028	Rural	75 ≤ x < 85	Incomplete elementary school	Female	Housemaid	4	Written

Separate analyses of the two groups produced two homologous sets of four interdependent themes: 1) *‘personal factors’*; 2) *‘external factors’*; 3) *‘clinical factors’*; and 4) *‘the HCP-patient-carer relationship’*. Tables 5 and 6 summarise coding for patient and carer participant groups, respectively. Due to maximum variation sampling, the frequency of a specific view is not indicative of the view’s importance, but rather its popularity and presence within the study sample [47].

**Table 5:** Patient themes representing factors influencing leprosy TOs

Theme	Facilitators of optimal TOs (frequency out of 14)	Barriers to optimal TOs (frequency out of 14)
**Personal factors**	▪ Belief in pharmaceutical treatment (11) ▪ Use of an education source (14) ▪ Health is important to the patient (12) ▪ High standard patient knowledge on resistance or the importance of compliance (13) ▪ Normal nature or lifestyle of participant did not change (4) ▪ Perceptions of leprosy as ‘dangerous’ or ‘contagious’ (10) ▪ Positive patient attitude, desire to be cured (13) ▪ Positive perceptions of the future, and hope in a cure (14) ▪ Psychological resilience (13) ▪ Witnessing another getting better or worse (7)	▪ Belief in traditional medicines (4) ▪ Belief medication is ‘strong’ (1) ▪ Change in the normal nature/appearance/lifestyle of patient (12) ▪ Change in identity, feeling labelled (8) ▪ Contradicting sources of education (11) ▪ Distrust of pharmaceutical medications (8) ▪ Experience/fear of stigma and discrimination (11) ▪ Feeling helpless (8) ▪ Isolation or distance (9) ▪ Myths and misinformation (12) ▪ Other comorbidities (6) ▪ Poor quality/limited knowledge (13) ▪ Psychological impact of leprosy (14)
**External factors**	▪ Ease of access to treatment (10) ▪ Education to empower social circle to help the patient/reduce stigma (9) ▪ Social circle support (emotional, nutritional, psychological, financial) (11) ▪ Family makes sacrifices for patient (6) ▪ Holistic care by HCP team (4) ▪ Individualised, patient-led care (5) ▪ Pragmatic approach to a high standard care in a resource scarce setting (1) ▪ Religion forms psychological support (10)	▪ Care is not holistic (5) ▪ Difficulty accessing treatment (5) ▪ Disorganised care (6) ▪ Social circle has poor/ limited knowledge of the disease (3) ▪ Financial impact (8) ▪ Impact of illness on social circle (social/psychological/financial) (11) ▪ Impact on aspirations for the future (5) ▪ Lack of political will to tackle leprosy (5) ▪ Living in the countryside away from services (2) ▪ Resource scarce health system (2) ▪ Impact on employment (12)
**Clinical factors**	▪ Contact tracing (4) ▪ Mental/physical preparation for treatment (10) ▪ Seeing/feeling improvement (10) ▪ Strategies reducing side effects (6)	▪ Late diagnosis (10) ▪ Long duration of treatment, high frequency doses (12) ▪ Comorbidities (6) ▪ Painful/distressing diagnosis (6) ▪ Progression or persistence of disease (14) ▪ Side effects of treatment (12)
**HCP-patient-carer relationship**	▪ Clear information provided (diagnosis, treatment, prognosis) (12) ▪ Good communication with HCP (9) ▪ Good quality care (6) ▪ Good, open, trusting HCP-patient-carer relationship (11) ▪ Patient feeling valued (7) ▪ Patient feels HCPs are important in their care (14)	▪ Clear information not provided on diagnosis, treatment and prognosis (11) ▪ Poor communication/relationship with HCP (4)

**Table 6:** Carer themes representing factors influencing leprosy TOs

Theme	Facilitators of optimal TOs (frequency out of 13)	Barriers to optimal TOs (frequency out of 13)
**Personal factors**	▪ Belief in pharmaceutical treatment (10) ▪ Use of an education source (12) ▪ Health is important to the patient (7) ▪ High standard patient knowledge on resistance or the importance of compliance (11) ▪ Normal nature or lifestyle of participant did not change (11) ▪ Perceptions of leprosy as ‘dangerous’ or ‘contagious’ (10) ▪ Positive patient attitude, desire to be cured (9) ▪ Positive perceptions of the future, and hope in a cure (13) ▪ Psychological resilience (7) ▪ Witnessing another getting better or worse (7)	▪ Belief medication is ‘strong’ (2) ▪ Change in the normal nature/appearance/lifestyle of patient (6) ▪ Change in identity, feeling labelled (1) ▪ Contradicting sources of education (7) ▪ Distrust of pharmaceutical medications (4) ▪ Experience/ fear of stigma and discrimination (3) ▪ Feeling helpless (1) ▪ Isolation or distance (4) ▪ Myths and misinformation (8) ▪ Other comorbidities (6) ▪ Poor quality/limited knowledge (11) ▪ Psychological impact of leprosy (11)
**External factors**	▪ Ease of access to treatment (14) ▪ Education to empower social circle to help the patient/reduce stigma (9) ▪ Social circle support (emotional, nutritional, psychological, financial) (11) ▪ Family makes sacrifices for the patient (6) ▪ Holistic care by HCP team (4) ▪ Individualised, patient-led care (5) ▪ Pragmatic approach to a high standard care in a resource scarce setting (1) ▪ Religion as a source of psychological support (10)	▪ Care is not holistic (2) ▪ Difficulty accessing treatment (4) ▪ Disorganised care (2) ▪ Financial impact (7) ▪ Impact of illness on social circle (social/psychological/financial) (5) ▪ Impact on aspirations for the future (4) ▪ Infected family members (3) ▪ Lack of conversation about leprosy with patient (4) ▪ Lack of political will to tackle leprosy (3) ▪ Living in the countryside away from services (2) ▪ Impact on employment (6)
**Clinical factors**	▪ Contact tracing (3) ▪ Mental/physical preparation for treatment (9) ▪ Seeing/feeling improvement (7) ▪ Strategies reducing side effects (5)	▪ Late diagnosis (3) ▪ Long duration of treatment, high frequency doses (11) ▪ Painful/distressing diagnosis (3) ▪ Progression or persistence of disease (9) ▪ Side effects of treatment (5)
**HCP-patient-carer relationship**	▪ Clear information provided (diagnosis, treatment, prognosis) (7) ▪ Good communication with HCP (6) ▪ Good quality care (7) ▪ Good, open, trusting HCP-patient-carer relationship (8) ▪ Patient feeling valued (3) ▪ Carer feels HCPs are important in their care (12)	▪ Clear information not provided on diagnosis, treatment and prognosis (4) ▪ Patient fear or experience of stigma or discrimination from HCP (1) ▪ Poor communication/relationship with HCP (4)

Quotes have been selected to aid understanding of themes and subthemes, and illustrate similarities and differences in the sample’s opinion.

### Theme 1: personal factors

This theme explores factors depending solely on the participant, and is subdivided into three subthemes: *‘knowledge and information quality’*; *‘health beliefs’*; and *‘psychological impact and character’*.

#### Subtheme a: knowledge and information quality

13 patients and 11 carers exhibited limited overall understanding of leprosy, with a sizeable proportion unable to provide even simple explanations about the disease, and almost all unable to identify the cause of leprosy transmission.

***Researcher:*** *‘What do you know about leprosy’?*

***Participant:*** *‘I have virtually no knowledge’* ***(018 – Patient)***

***Researcher:*** *‘How do you think you get the disease?’*

***Participant:*** *‘I never shower with hot water and then suddenly with cold, or after eating, because I thought that’s what causes leprosy. I don’t know how I got it.’* ***(018 – Patient)***

***Participant:*** *‘Because of cockroaches.’* ***(019 – Patient)***

***Participant:*** ‘*The sun. Petrolina is hot, and [the patient] was out at midday.’* ***(020 – Carer)***

Despite all participants either experiencing leprosy relapse, or caring for a relapse patient, relapse was poorly understood in all but one participant. Many patients and carers did not realise that the treatment they/the patient was receiving was a separate, second course of medications, targeting relapsed leprosy. Some patients perceived health education as their HCPs’ responsibility, and consequently blamed their poor relapse knowledge on inadequate HCP-led education.

***Researcher:*** *‘Do you know what relapse is?’*

***Participant:*** *‘No, sorry. Nobody at the clinic ever told me about that.’* ***(009 – Patient)***

***Participant:*** *‘ … my sister-in-law said it was when her leprosy came back.’* ***(017 – Carer)***

Some carers had poor knowledge because they perceived the patient as the primary knowledge source, and did not use other accessible means to obtain further information. Carers with good knowledge were proactive and resourceful when searching for information.

***Participant:*** *‘We are curious, but [the patient] never told me about leprosy, so I never asked.’*

***Researcher:*** *‘Did you look online or in health centres instead?’*

***Participant:*** *‘No.’* ***(013 – Carer)***

***Participant:*** *‘I like to learn more about leprosy so I can take better care of [the patient].’* ***(003 – Carer)***

More knowledgeable participants agreed that the internet (for those with access) and television programmes, were useful, information-rich sources supplementing HCP-given knowledge between clinic visits. Programmes dedicating airtime to Brazil’s national leprosy campaign, *‘Purple January’*, effectively raised public awareness [63]. Some participants expressed the importance of mass, societal education on leprosy, and alluded to schools being an effective medium.

***Participant: ‘****After the appointment I searched the internet and learnt a lot of things.’*

***Researcher:*** *‘Were you more relieved when you had more information?’*

***Participant:*** *‘I was … when we don’t know things, it feels like a shock, right?’* ***(001 – Patient)***

***Participant:*** *‘The thing about ‘Purple January’ is that people talk about it. It is necessary for school to teach about leprosy. These campaigns are needed.’* ***(026 – Patient)***

Some felt that ‘word of mouth’ knowledge was less reliable than HCP-led education, which was greatly valued. Others gained rich, anecdotal knowledge from ‘expert patients’.

***Participant:*** *‘People get in the way, saying this, saying that. And [the patient] just gets confused. But the clinic doctor, she studied for this, she knows what is right and wrong.’* ***(023 – Carer)***

***Participant:*** *‘ … I know the woman in the waiting room. She told me about her nerves. They became defective. Others I know are on crutches, they can’t walk.’* ***(001 – Patient)***

Some participants were familiar with leprosy’s biblical associations [63]. However, these participants agreed that the Bible is an unreliable clinical resource.

***Participant:*** *‘I read about leprosy, about Job, in the Bible, where it is a chronic disease and difficult to treat. Job had itchy wounds but there was no treatment then. Now it’s different.’* ***(006 – Carer)***

#### Subtheme B: health beliefs

Many perceived leprosy as dangerous or contagious, posing threat to health. Consequently, when asked to hypothesise about why some patients may have poor medication compliance, these participants felt this was due to a poor regard for personal health.

***Participant:*** *‘It’s contagious, and it causes numbness, which is scary, because you cut yourself but can’t feel it.’* ***(017 – Carer)***

***Participant:*** *‘[Patients who are non-compliant] don’t want to be healed.’* ***(016 – Patient)***

Carers believed their health had elevated importance, due to their patient responsibilities.

***Participant:*** *‘The importance of my health is everything because … I dedicate myself to [the patient].’* ***(003 – Patient)***

Most participants felt positively towards pharmaceutical leprosy medications. Participants who witnessed their own or another’s leprosy improve more commonly expressed belief in medications.

***Participant:*** *‘Because others have healed, why won’t I?’* ***(022 – Patient)***

Conversely, those yet to witness symptomatic improvements expressed medication distrust. Despite this, all patients stated they were wholly compliant with their medication regime.

***Participant:*** *‘People stop the medicines because it takes time to see effects. They don't believe in them because they give up before getting better.’* ***(003 – Carer)***

***Participant:*** *‘I could have stopped taking the pills. But the clinic staff kept saying, "Don't stop, otherwise it comes back even worse.". So, I kept going.’* ***(010 – Patient)***

Some participants, despite perceiving the medication positively, did not believe the medication was curative. Some justified this belief by explaining that the medication could not possibly treat severely advanced leprosy, but only alleviate symptoms.

***Participant: ‘****I want them to find a cure because these remedies, they combat, but don’t cure.’* ***(003 – Carer)***

***Participant:*** *‘I think that at my stage, I don’t know if I will be cured. It cures you only if you have a few lesions.’* ***(027 – Patient)***

Some people incorrectly believed that their medication side effects were instead a sign of their leprosy prognosis worsening. Numerous labelled pharmaceutical medications as *‘strong’*. Some hypothesised about the effectiveness of *‘natural’* remedies, perceived as *‘safer’*.

***Participant:*** *‘All these new problems, the medicine isn’t working because it gave me new problems.’* ***(004 – Patient)***

***Participant:*** *‘I only take the medicines with food. They are so strong, they attack the stomach, kidneys, liver.’* ***(019 – Patient)***

***Participant:*** *‘Maybe certain vegetables, or maybe herbal medicines [cure leprosy]? Would you know?’* ***(026 – Patient)***

When asked about the advice they would give to newly diagnosed leprosy patients, despite some participants expressing treatment disbelief, almost all stressed the importance of medication compliance.

***Researcher:*** *‘What advice would you give to a newly diagnosed leprosy patient?’*

***Participant:*** *‘Follow the treatment correctly so it doesn’t come back and gets better.’* ***(015 – Carer)***

***Participant:*** *‘It’s a delicate treatment that has to be treated according to the doctor, so follow their advice.’* ***(002 – Patient)***

#### Subtheme C: psychological impact and character

All patients and 11 carers described leprosy’s significant psychological impact. Most described this impact as prolonged, spanning from the time of symptom appearance and persisting indefinitely. Numerous patients felt they were no longer *‘normal’*, and felt labelled as *‘sick’*. Visual leprosy manifestations affected self-esteem, particularly in females. Participants expressed desperation for a cure.

***Participant:*** *‘My beautiful legs, my lovely feet, suddenly looked bruised … If there is no cure, I will jump off a bridge. Because I will not live life sick with this leprosy, like a loser. I just want to be the same as I was.’* ***(018 – Patient)***

***Participant:*** *‘It did [have a psychological impact]. For two months, I wouldn’t walk with anyone, only alone or in the night. I didn’t sleep. I didn’t realise, but I would go to shower and still have all my clothes on. I was upset, dejected.’* ***(018 – Patient)***

This altered sense of identity was augmented by the impact leprosy had on the roles and responsibilities of participants; due to issues surrounding their ability to work, disease transmission fears, and dependency on others.

***Researcher:*** *‘Do you think leprosy has a big impact on your life?’*

***Participant:*** *‘Yes. Because I use a crutch, I have to be with someone when I go out so they can help. It’s annoying. The pain means I don’t sleep well, and I don’t have a normal day like others.’* ***(010 – Patient)***

Nine patients and four carers felt leprosy caused isolation and distance, causing further psychological impact. Isolation from close family members had the greatest psychological impact.

***Participant:*** *‘My husband was scared. He was afraid of having sex … my daughter said, “Mom, if you have leprosy, you have to separate.”. She separated her glass, her plate, everything.’* ***(026 – Patient)***

Eleven patients, compared to only three carers, reported experiences of stigma. This shows a lack of conversation about stigma between the two subgroups. Several participants reporting stigma were parents of infected school-going children. Leprosy’s biblical links, widely recognised in Brazil’s large Catholic population, [64, 65] caused experiences of shame and self-stigma.

***Participant:*** *‘Someone I worked with asked me why I was dark. I said it was because of leprosy treatment. She stepped back. She welcomed me with a kiss. But once she knew it was leprosy, she was quick to leave.’* ***(026 – Patient)***

***Participant:*** *‘They said it is not a disease of people, but of animals.’* ***(012 – Patient)***

***Participant:*** *‘My son faced stigma. Some kids found out at school and made fun of his colour and called him names.’* ***(025 – Carer)***

However, almost all participants emphasised the importance of a positive patient outlook. Both groups recognised negative behaviours enhancing leprosy’s psychological impact, and agreed this could be combatted by a desire to be cured and optimistic attitudes. Many carers spoke of keeping patients positive and motivated towards being cured.

***Participant:*** *‘I didn’t speak to anyone for a month. Then my family told me being isolated is worse … you have to talk with your loved ones.”.’* ***(002 – Patient)***

***Participant:*** *‘I took the medication just how the doctor said. I gave myself every chance to get better.’* ***(005 – Patient)***

***Participant:*** *‘He never stopped the medicine because he just wanted to get better, get back to work. I always said, “Don’t give up!”, because otherwise there is no point.’* ***(003 – Carer)***

Six patients mentioned difficulties managing comorbidities alongside leprosy (namely aging, hypertension, alcoholism/drug use, mental health illness, obesity and diabetes), which resulted in new or worsening psychological symptoms. Further issues surrounding polypharmacy, worsening general health and poor treatment regimen commitment caused patients *‘distress’*.

***Participant:*** ‘Leprosy made everything worse. It made me so upset. I had to close my business. Then I was even more upset so I started drinking cachaça* even more, smoking marijuana … ’ ***(018 – Patient)***

*Cachaça: a distilled spirit popular in Brazil

***Participant:*** *‘I have diabetes. I think the treatment made it worse. When I stopped taking the medicine, the doctor said my diabetes got better.’* ***(027 – Patient)***

### Theme 2: external factors

External factors describe factors outside of patient control. This theme is subdivided into three subthemes: *‘socioeconomic factors’*; *‘structural factors’*; and *‘support factors’*.

#### Subtheme a: socioeconomic factors

Leprosy’s socioeconomic impact was mentioned by all patients and most carers. Eight patients and seven carers felt leprosy financially impacted them, as their ability to work was restricted by symptoms and side effects, or carer responsibilities. Wider financial implications concerned rent payments, costs travelling to clinics, and household/family costs. One patient explained that ‘*Bolsa Familia’*, a Brazilian social welfare programme providing financial aid to poor families on conditions that their children attend school and receive vaccinations, partly supplemented lost earnings [66].

***Participant:*** *‘I worked in grape farms. Today, I don’t have the courage to work anymore, under the hot sun … I am too weak. But if I don’t go to the farm, how will I feed my children? Their lives will suffer.’* ***(001 – Patient)***

***Participant:*** *‘I can’t work … I have to care for [the patient].’* ***(003 – Carer)***

***Participant:*** *‘I got Bolsa Familia, because I couldn’t work, right?’* ***(003 – Carer)***

Younger patient participants felt leprosy impacted them financially because their career aspirations were affected, as they expected to face a lifetime of stigma in working environments. Some felt these implications were exacerbated by local job shortages.

***Participant:*** *‘Who will hire a person with leprosy? There is prejudice. I need to heal. I need to be cured. Because I have dreams, projects … ’* ***(026 – Patient)***

***Participant:*** *‘Petrolina is a very bad place to get a job. He paid for expensive colleges and never got a job.’* ***(028 – Carer)***

#### Subtheme B: structural factors

Structural factors concern organisational, regional and national level issues affecting patient care. While four patients and six carers felt care was holistic and individualised, numerous disagreed. Participants felt there was insufficient regional investment in services addressing psychological needs of leprosy patients.

***Participant:*** *‘I told the doctor I felt very weak. She did all the tests quickly, found out I was anaemic and gave me medicines for it.’* ***(018 – Patient)***

***Participant:*** *‘The doctor does counselling, but there should be a psychologist. Petrolina has to invest more in this.’* ***(005 – Patient)***

Six patients and two carers felt the health care system was disorganised, consequently negatively impacted their perceptions of care. Two patients noted medication and healthcare equipment resource scarcity in clinics, with one participant explaining that rural clinics had an additional lack of specialised staff. Some participants blamed this on the government’s poor national and regional healthcare decision-making. One carer felt that despite this, resources were pragmatically distributed.

***Participant:*** *‘Someone did a blood test last year. We never heard back. The results weren’t here, so it looks like they were lost.’* ***(020 – Carer)***

***Participant:*** *‘So, at my clinic, there was no health agent, no doctor … ’* ***(019 – Patient)***

***Participant:*** *‘Previously it was difficult but nowadays if one health unit doesn't have treatment, they bring it from another unit. If I run out [of medication] and they don’t have it, I just try again the next day.’* ***(003 – Carer)***

***Participant:*** *‘The medication is made abroad; I think that is why sometimes there is a lack of medication. It depends on the government, but I don’t think they take public health seriously in Brazil.’* ***(006 – Carer)***

***Participant:*** *‘It is difficult in the countryside. There is a lack of awareness. Health care professionals need to visit us at home because it is hard travelling to clinics.’* ***(005 – Patient)***

#### Subtheme C: support factors

HCP support was reported by all participants. This came in the form of imparting knowledge, prescribing medication, and providing strategies to make side effects tolerable.

***Participant:*** *‘[The HCPs] helped me a lot. In everything. In giving advice, explaining things … ’* ***(010 – Patient)***

Participants felt family support was *‘vital’*. While both groups recognised leprosy impacted family and societal relationships, due to stigma, transmission risk, and dependency, both groups also credited their support. Patients and carers with less dialogue between them about leprosy reported less intimate, unreliable support provision. Conversely, open patients who educated their carers about leprosy empowered them to provide individualised support.

***Participant:*** *‘Only my eldest boy works. I have another boy at school. He needs me as a mother, but I am not well.’* ***(019 – Patient)***

***Participant:*** *My neighbour said, "Get away from him, that disease is transmissible!". So, I isolated myself, but then, when his wife and children came out to talk to me, I told them, "No, I am not infectious.".* ***(002 – Patient)***

Support from the family and social circle came in emotional, nutritional, psychological and financial forms.

***Participant:*** *‘People asked why [the patient] didn’t leave the house. I said that he was ashamed of his leprosy. He had depression. So, I said, "Since you won’t go out, I’ll invite everyone home.".’* ***(003 – Carer)***

***Researcher:*** *‘What is your role in the patient's life?’*

***Participant:*** *‘Her friend, her counsellor, her helper, her strength … ’* ***(025 – Carer)***

***Participant:*** *‘My sister, she pays for my water, my light. She makes food for my house when I can’t do it.’* ***(019 – Patient)***

Participants in both groups used religion as another form of psychological support.

***Participant:*** *‘I believe he will be cured. I believe in God and whoever believes in God has everything. You have to have faith. There are days when he at home is agonized, restless. I take the Bible and read to him.’* ***(024 – Carer)***

### Theme 3: clinical factors

Clinical factors refer to a patient’s healthcare necessities. This theme is subdivided into two subthemes: *‘treatment and side effects’*, and *‘experiences of diagnosis’*.

#### Subtheme a: treatment and side effects

Twelve patients and five carers experienced medication side effects. The most frequently occurring side effect was sunburn; due to photosensitivity reactions associated with multidrug therapy [67]. Other commonly reported side effects were headache, gastrointestinal problems, and weakness. These side effects reduced treatment adherence. Participants worked with HCPs to find strategies to overcome side effects, making the medications more agreeable to patients.

***Participant:*** *‘It’s hot here every day, and I’m exposed to the sun because I’m a farmer, so my skin became dark because of the medication.’* ***(019 – Patient)***

***Participant:*** ‘I kept vomiting when I swallowed the pills, so the doctor gave me [dimenhydrinate*].’ ***(010 – Patient)***

*Dimenhydrinate: an antihistamine medication used to prevent nausea and vomiting.

***Participant:*** *‘She became weak. They said she had anaemia because of the medicines so the nurse gave her ferrous sulfate.’* ***(025 – Carer)***

***Participant:*** *‘When you take it in the morning you feel that nausea, right? But when you take it before sleep, you don’t feel anything.’* ***(026 – Patient)***

All participants commented on the long treatment regimen and dose frequency. Many felt that being mentally prepared to take the medication was important in remaining compliant. Carers encouraged patient compliance. The majority either saw or felt improvement with medication, while some, who did not, felt less motivated to stay compliant.

***Participant:*** *‘The treatment has so many medications, so many pills every day.’* ***(007 – Carer)***

***Participant:*** *‘Since the disease has a cure, you have to try to do everything to make things better.’* ***(016 – Patient)***

***Participant:*** *‘She told me she had to restart treatment, and the only thing I said was, "Do it, don't stop, continue to the end, take the steps you have to take".’* ***(017 – Carer)***

***Participant:*** *‘From the second day onwards, it was only improvement.’* ***(012 – Patient)***

***Participant:*** *‘I have already been treated for two years and I still have the leprosy. Where is there result? I wanted to stop taking the medicines, but the clinic staff said that wouldn’t be good.’* ***(010 – Patient)***

#### Subtheme B: experiences of diagnosis

10 patients recalled a delayed diagnosis. For some, this was due to receiving initially incorrect diagnoses, which left patients *‘distressed’*. For others, this was because they did not seek HCP advice until their symptoms significantly progressed. Only four patients and three carers recalled household contact tracing. Through these responses, it became apparent that contact tracing was not only uncommon, but, when done, too infrequent to be effective [68].

***Researcher:*** *‘How long after seeing the first lesion did you wait before seeing a doctor?’*

***Participant:*** *‘A year or so passed. I went to the doctor when I became numb.’* ***(010 – Patient)***

***Participant:*** *‘The hospital told me it was rheumatism. It got worse so I went to the clinic again. Then they told me I had advanced leprosy. If they told me sooner, maybe I would have suffered less.’* ***(004 – Patient)***

***Researcher:*** *‘When [the patient] was diagnosed, were you examined?’*

***Participant:*** *‘No. Not me nor my children, no one. I didn’t think about [contact tracing] until you just said.’* ***(008 – Carer)***

***Participant:*** *‘The health professionals were concerned not only about me, but also my family. To prevent the disease, right?’* ***(017 – Carer)***

### Theme 4: HCP-patient-carer relationship

This theme explores how the relationship between HCPs, patients and carers impacted TOs.

All participants felt HCPs were vital to their care, and recognised the importance of strong HCP-patient-carer relationships. This relationship served as the foundation for good communication and trust. While nine patients and six carers felt that communication was good with HCPs, others disagreed. The clarity of information provided by HCPs similarly received mixed reviews, leading several to feel the medication regime was *‘complicated’*. Many reported that HCPs simply stressed the importance of treatment compliance, but did not offer deeper information.

***Participant:*** *‘I learned a lot from the doctor. She said there are five types of leprosy, and mine attacks the nerves and causes me to have reactions.’* ***(002 – Patient)***

***Participant:*** *‘The doctor said I have to take the medicine every day without fail, right?’*

***Researcher:*** *‘Did they tell you about the side effects, the reason why you need it, anything like that?’*

***Participant:*** *‘No.’* ***(001 – Patient)***

***Participant:*** *‘I was surprised. How come the doctor stopped the medication if I still had a lesion? It bothers me a lot that I stopped the treatment then. Nobody told me exactly why.’* ***(019 – Patient)***

Opinions were also mixed concerning the trust between HCPs and participants. Some participants suspected HCPs of omitting important information during consultations. For others, outcomes desired by patients appeared misaligned with those desired by HCPs. The interdependence of trust and good communication becomes apparent through such responses.

***Participant:*** *‘The doctor at my clinic, I tell her everything. My whole story of suffering, everything.’* ***(019 – Patient)***

***Participant:*** *‘Maybe, the doctors don’t tell me about everything I should know.’* ***(006 – Carer)***

***Participant:*** *‘In the eyes of the doctors, I am better, but my leg still feels numb, that’s the problem.’* ***(002 – Patient)***

Most participants, however, felt comfortable sharing worries with HCPs. A number mentioned specific staff members who were exceptionally helpful in their care. Participants felt valued by HCPs, and respected in the clinical environment.

***Participant:*** *‘I was worried about my daughter. The doctor told me, “Look, she will live a normal life, study, date, everything.”. I felt relieved.’* ***(025 – Carer)***

***Participant:*** *‘I am always welcomed in the clinic. All the staff take good care of patients; they are very polite, very excellent indeed.’* ***(003 – Carer)***

***Participant:*** *‘The two doctors here are very good. If the doctors are worried about your health, they will find out what is wrong as soon as they can.’* ***(026 – Patient)***

## Discussion

This study explored factors influencing leprosy TOs through interviews with 27 leprosy retreatment patients and carers. Separately analysing the two groups produced two homologous sets of four interdependent themes: *‘personal factors’*; *‘external factors’*; *‘clinical factors’*; and *‘the HCP-patient-carer relationship’*. These themes provide a comprehensive societal insight on TOs, and collectively challenge current leprosy management strategies in Pernambuco, Brazil.

### Personal factors

The study findings suggest that the psychological effects of leprosy, health beliefs, quality and extent of knowledge and character impact leprosy TOs.

Both participant groups displayed poor basic knowledge of leprosy. Poor knowledge on the cause of leprosy facilitated distrust in pharmaceutical medications, as this knowledge is required to appreciate that antimicrobials combat infection [69]. This pharmaceutical medication distrust increased likelihood of belief in non-evidence based, traditional medicines, which themselves have poorer TOs [70, 71]. Poor knowledge about relapse also fostered pharmaceutical medication distrust. Most participants did not distinguish their/the patient’s current treatment as a separate, second round of medication for relapse; facilitating perceptions that the medication regimen is unnecessarily long, ineffective and a source of unwanted side-effects. Poor knowledge about leprosy transmission formed another barrier to optimal TOs; reducing preventative and symptom-presenting behaviours [72]. Improving the study population’s basic knowledge of leprosy and treatment may increase pharmaceutical medication belief, and facilitate psychological coping with treatment length and side effects. Additionally, countering false transmission beliefs with evidence-based knowledge may dampen Pernambuco’s *‘hyperendemic’* status, by encouraging the public to: seek contact tracing; be wary of transmission routes; and understand how treatment compliance makes transmission improbable [73–75]. This relationship between disease knowledge and positive TOs is supported by literature studying other diseases [27, 76–81].

HCPs were perceived as the most superior knowledge source, effectively endorsing treatment compliance, including in those expressing medication distrust. Future strategies should exploit this population’s faith in HCPs to increase leprosy TOs, by a) maximising HCP-led leprosy education towards patients and carers, and b) encouraging HCPs to increase public health-seeking behaviour, through sign-posting other reliable, easily accessible resources. HCP-led leprosy education could tackle Pernambuco’s financial burden of avoidable ‘treatment drop-out’ and relapse patients, as literature on other diseases suggests this as a cost-effective method on larger, policy-making scales [19, 82–84]. Agreeing with previous literature, [85], ‘expert patients’ were perceived as valuable supplementary knowledge source. This study promotes using ‘expert patients’ to increase societal leprosy awareness, and spread positive experiences to patients and carers yet to witness symptomatic improvements (a subgroup more likely to develop pharmaceutical medication disbelief).

This study highlights leprosy’s relationship with psychological wellbeing. The transition of family members into carers altered relationship dynamics, due to changes in physical and emotional dependency levels, intimacy and financial support. The roles and responsibilities of patients and carers changed, with occupation almost always impacted. This caused altered/lost identities in both patients and carers. Psychological comorbidities, external and self-stigma (fuelled by societal perceptions of leprosy as *‘dangerous’* and biblical associations), and visual symptoms (particularly in females), exacerbated psychological distress [64]. Given the impact of mental wellbeing on TOs, [86–88] these findings promote a psychological component in leprosy treatment, led by mental health specialists trained to provide individualised care [89]. Strategies to protect psychological wellbeing, such as mental preparation for treatment and maintaining positive outlooks, could additionally be promoted by HCPs to newly diagnosed patients and their carers, to aid coping. Campaigns promoting leprosy as curable and non-transmissible during treatment may reduce existing societal stigma, further reducing leprosy’s psychological impact [90, 91]. Executing such campaigns on mass scales could target all pockets of society, with widespread positive implications.

### External factors

The study findings also indicate that socioeconomic, structural and support factors impact leprosy TOs.

Leprosy exhibited several negative, interdepending, socioeconomic consequences on patients and carers. Increases in disease and side effect severity made patients increasingly dependent on carers; impacting both groups’ abilities to maintain employment. Participants implied that unless employment loss was compensated for by carers adopting additional earning responsibilities, household ability to afford rent, nutritious food, and travel to appointments would be thwarted. Both of these consequences decrease patient treatment engagement, as either patient care is compromised, or patients progressively lose wealth and become impoverished. This results in a vicious cycle of increasing dependency and consequent financial instability [92–97]. While Brazil’s *‘Bolsa Familia’* conditional cash transfer programme aims to protect from this vicious cycle, [98, 99] it is perhaps inadequate as income replacement; because while several participants alluded to their household financial insecurity, only one felt supported by *‘Bolsa Familia’*. Participants with aspiring careers or infected children were not immune to this vicious cycle; as stigma in occupational and educational settings, alongside Pernambuco’s job shortages and poverty [18, 46], impacted pursuing vocational education and training. This study promotes local financial support schemes for leprosy patients and carers, to shield from continuous financial setbacks. Food vouchers in other Brazilian states and direct income replacement in Nigeria and China have shown promise, and could be trialled in Pernambuco [100–102]. Superior to financial or material help, vocational skills and disability-friendly workplaces may enable patients and carers to permanently escape this socioeconomic vicious cycle, improving TOs [103].

This study exposes regional resource scarcity and healthcare disorganisation, particularly rurally. Possibly a direct consequence of Brazil’s geographical healthcare inequalities, these findings underline a need for regional and national changes in: healthcare quality standards; resource distribution; and public healthcare investment [15]. The several avoidable retreatment patients in Pernambuco present unnecessary financial burdens to healthcare policy makers [19]. Investing into improved resources and holistic care may be a cost-effective long-term solution; as the resulting improved TOs will generate smaller future expenditures on leprosy patients, and a larger, fit-to-work, economy-contributing population [104].

While HCP support is well recognised in wider literature**,** this study provides novel insight on carer roles [105, 106]. Family members adopted carer responsibilities for patients; overcoming personal stigmas and transmission fears to provide psychological, nutritional, and financial support. Some patients became their carers’ personal ‘expert patient’; fostering stronger, individualised support-giving, consequently facilitating TOs. Although modifying patient motivation and attitude is inherently challenging, as behavioural change is complex and multifactorial, [107] this study shows carer-led encouragement increases patient psychological wellbeing and resilience. Encouraging conversations about stigma between patients and carers may further nurture trusting, open relationships; supplementing carer-led psychological support [91]. HCPs could utilise their trustworthiness to encourage more intimate patient-carer dialogue. Despite leprosy’s biblical associations fuelling self-stigma for some [64], religion provided personal support mechanisms facilitating optimal TOs for many. Once religion is identified as a supportive source for an individual, HCPs could further improve psychological wellbeing (and consequently TOs), by directing patients and carers to religious congregations and worship places.

### Clinical factors

This study and previous literature appreciate that treatment side effects negatively impact TOs [32, 100, 108]. This study additionally supports side effect-reduction strategies (using additional medication or modifying medication administration times) to facilitate TOs. Echoing previous literature findings, lengthy treatments and medication ingestion frequency, both inflexible, also negatively affected TOs, particularly in polypharmacy patients [27, 32–36]. However, mental resilience and preparedness made treatment lengths and dose frequency more agreeable to patients. This is perhaps because such individuals had better medication compliance, and consequently witnessed symptom improvement, which confirmed belief in treatment. This study also found that delayed diagnosis, due to incorrect HCP symptom recognition, poor health-seeking behaviours, and absent/infrequent (hence ineffective) contact tracing, negatively affected TOs.

HCPs, as the gateway to healthcare delivery, are key to facilitating optimal TOs clinically [34]. The study findings suggest HCPs should reassure patients that strategies to overcome side effects exist, promote psychological resilience, and endorse awareness campaigns like ‘Purple January’ to facilitate TOs [63]. HCP retraining on symptom awareness, contact tracing and evolving MRI/ultrasonography modalities may aid earlier disease diagnosis and improved TOs, as shown in wider literature [109, 110]. Observations from HCP retraining workshops held at the time of the study (Fonseca A, personal communication, March 2020); alongside findings of a simultaneously conducted study on HCP perceptions towards leprosy by co-author MB, indicate urgency for better location-specific HCP retraining and patient education [111].

### HCP-patient-carer relationship

Resonating with previous literature, good patient/carer relationships with HCPs facilitated trust, and consequently improved TOs [27, 32–36]. Patients and carers credited HCPs as ‘vital’ for care, and felt valued, welcomed and comfortable sharing concerns with them. Some staff members were recognised numerous times for their care quality. However, this study also exposes a need for improved depth and clarity of HCP-led communication. Patients practiced treatment compliance despite HCPs providing dissatisfactory, surface-level communication at times. This points towards paternalistic medicine cultures, presenting additional TO barriers [100, 112]. Increasing dialogue and trust between HCPs, patients and carers, through HCP communication skills retraining, may encourage more satisfying, individualised care experiences [100, 112]. This study stresses the importance of cultivating strong carer-HCP relationships. Encouraging carers to attend appointments will enable them to interpret HCP advice with patients, developing the carer role into one of even higher quality individualised care.

### Strengths and limitations

This study’s primary strength is the deep insight it provides on the perceptions, experiences and beliefs of leprosy and treatment in Pernambuco, a ‘*hyperendemic*’ yet under-researched location. However, given this study’s qualitative design, caution should be taken when generalising findings to populations beyond those explored [113]. Additionally, the use of small participant numbers, and exclusion of ‘treatment dropout’ patients (due to recruitment difficulties), impact representativeness. This deserves appreciation before applying these findings to similarly endemic locations [49].

As a cross-language study, translators and interpreters impacted data collection and analysis [114]. Braun and Clarke’s method obtains meanings from collected textual data [56]. Therefore, interpreters and translators increase chance of obtained meanings becoming dissimilar to those actually expressed by participants, by being ‘lost in translations’ [114]. Wider literature, however, suggests that thematically analysing translated data largely does not impact thematic synthesis [115]. Strategies reducing bias relating to misinterpretation, involving pilot interviews and secondary, independent translation checks, were undertaken regardless. LD and PR, both Brazilian nationals, facilitated participant comfort during interviews, as they interviewed members of their own community; and clarified cultural references in participant responses. DK, PR and LD were neutral and uninvolved in patient care, however may have still evoked some social-acceptability bias.

In retrospect, further limitations exist in the lack of real-time translation during interviews. This would have allowed DK to delve deeper into desired topics, particularly surrounding participants’ thoughts on governmental and regional strategies for leprosy eradication, and religious beliefs.

Finally, the impact of DK, the lead researcher (British Indian, female medical student), conducting this cross-cultural investigation requires appreciation. Culture is outlined as a set of distinguishing, discriminating features of a social group [116]; therefore, as a foreigner to this community in Petrolina, DK’s presuppositions and beliefs may have biased data interpretation and analysis. These effects, and therefore researcher bias, were minimised through LD and PR explaining cultural references, triangulation and discussions on theme formation.

## Conclusion

This study identifies personal factors, external factors, clinical factors and factors relating to the HCP-patient-carer relationship impacting leprosy TOs. This study contributes to location-specific, state and national levels by informing the development of higher quality health promotion; holistic, individualised care provision; and evenly geographically distributed financial investment into leprosy patients and the healthcare provisions they utilise. Failure to address these findings will hinder regional elimination efforts. Further studies, exploring views of the ‘treatment dropout’ subgroup and evaluating the effectiveness of future, newly imposed interventions, may provide additional, highly relevant evidence.

## Supplementary Information

**Additional file 1.** TOPIC GUIDE FOR PARTICIPANTS. This file contains details of the topic guides used for interviewing the participants. The first table presents the topic guide for patient participants, while the second table presents the topic guide for carer participants.

## Data Availability

The datasets used and analysed during the current study are available from the corresponding author on reasonable request.

## Abbreviations

TO: Treatment outcome
HCP: Healthcare professional
MDT: Multidrug therapy
WHO: World Health Organisation
AMR: Antimicrobial resistance
NE: North East
SR: Systematic review
