# Supplementary material for: Improving treatment outcomes for leprosy in Pernambuco, Brazil: a qualitative study exploring the experiences and perceptions of retreatment patients and their carers
Source: BMC Infect Dis. 2021 Mar 19;21:282. doi: 10.1186/s12879-021-05980-5 (PMC7980336; doi:10.1186/s12879-021-05980-5)
Supplement: Supplementary file 1 — Additional file 1. TOPIC GUIDE FOR PARTICIPANTS. This file contains details of the topic guides used for interviewing the participants. The first table presents the topic guide for patient participants, while the second table presents the topic guide for carer participants. [file 12879_2021_5980_MOESM1_ESM.docx]

**Additional file 1: Topic guide for participants**

| **Table 1: Topic guide for patient participants** | |
| --- | --- |
| Topic | Subtopics |
| Questions exploring personal factors | - Knowledge about leprosy and treatment - Beliefs about leprosy transmission and cure - Experiences of restarting treatment - Importance of health - Leprosy manifestations - Medication side effects - Medication compliance and coping mechanisms - Psychological impact |
| Questions exploring external factors | - Employment and future aspirations - Experiences of diagnosis - Access to healthcare - Importance of HCP - Contact tracing |
| Questions exploring support network | - Sharing diagnosis with community - Stigma - Family and social circle - Religion |

| **Table 2: Topic guide for carer participants** | |
| --- | --- |
| Topic | Subtopics |
| Questions exploring personal factors | - Knowledge about leprosy and treatment - Beliefs about leprosy transmission and cure - Experiences of patient restarting treatment - Importance of health - Leprosy manifestations in the patient - Medication side effects and coping mechanisms - Medication compliance - Psychological impact on self and carer |
| Questions exploring external factors | - Experiences of carer role - Employment and future aspirations - Access to healthcare - Importance of HCP - Contact tracing |
| Questions exploring support network | - Sharing diagnosis with community - Stigma - Family and social circle - Religion |
